# Evaluation of aggressive posterior retinopathy of prematurity (AP-ROP) in the German Retina.net ROP registry

**DOI:** 10.3389/fmed.2026.1772424

**Published:** 2026-03-23

**Authors:** Marie-Christine Bründer, Johanna M. Pfeil, Marlene Sassmannshausen, Navid Farassat, Birgit Lorenz, Lyubomyr Lytvynchuk, Helge Breuss, Teresa Barth, Christos Skevas, Karsten Hufendiek, Tim U. Krohne, Daniela Suesskind, Anna Nguyen-Höhl, Marian Liegl, Barbara Glitz, Ameli Gabel-Pfisterer, Stefanie Gniesmer, Vinodh Kakkassery, Sabine Baumgarten, Rainer Guthoff, Lars Choritz, Andreas Stahl, German Retina.net ROP Registry Study Group

**Affiliations:** 1 Department of Ophthalmology, University Medicine Greifswald, Greifswald, Germany; 2 Department of Ophthalmology, University Medicine Bonn, Bonn, Germany; 3 Eye Center, Medical Center, Faculty of Medicine, University of Freiburg, Freiburg, Germany; 4 Department of Ophthalmology, Eye Clinic, Justus-Liebig-University Giessen, University Hospital Giessen and Marburg GmbH, Campus Giessen, Giessen, Germany; 5 Department of Ophthalmology, HELIOS Klinikum Berlin-Buch, Berlin, Germany; 6 Department of Ophthalmology, University of Regensburg, Regensburg, Germany; 7 Department of Ophthalmology, University Medical Center Hamburg Eppendorf, Hamburg, Germany; 8 University Eye Hospital, Hannover Medical School, Hannover, Germany; 9 Department of Ophthalmology, Faculty of Medicine and University Hospital Cologne, University of Cologne, Cologne, Germany; 10 University Eye Hospital, Eberhard Karls University of Tuebingen, Tuebingen, Germany; 11 Department of Ophthalmology, University Medical Center Goettingen, Goettingen, Germany; 12 Department of Ophthalmology, Vivantes Klinikum Neukoelln, Berlin, Germany; 13 Department of Ophthalmology, University of Muenster Medical Center, Muenster, Germany; 14 Department of Ophthalmology, Klinikum Ernst von Bergmann, Potsdam, Germany; 15 Department of Ophthalmology, University of Luebeck, Luebeck, Germany; 16 Department of Ophthalmology, Klinikum Chemnitz gGmbH, Chemnitz, Germany; 17 Department of Ophthalmology, University Hospital RWTH, Aachen, Germany; 18 Department of Ophthalmology, Faculty of Medicine, University of Duesseldorf, Duesseldorf, Germany; 19 Department of Ophthalmology, Otto von Guericke University, Magdeburg, Germany

**Keywords:** AP-ROP, A-ROP, retinopathy of prematurity, risk factors, ROP

## Abstract

**Purpose:**

Aggressive (posterior) retinopathy of prematurity (A-ROP/AP-ROP) may progress rapidly into tractional retinal detachment if not treated timely. Fortunately, this type of treatment-warranted ROP is rare in developed countries. As a consequence, data on risk factors for development of this aggressive form of ROP is sparse. In this study, we aim to analyse the risk factors for the development of AP-ROP using data from the German Retina.net ROP registry and report functional outcomes of patients treated for AP-ROP.

**Methods:**

We assessed the observational German Retina.net ROP registry for infants born between 2011 and 2020 and treated for unilateral or bilateral aggressive posterior ROP (AP-ROP) (*N* = 21). These infants were compared to infants treated for ROP without AP-ROP (control group; *N* = 329) regarding demographics, neonatal parameters, ROP management and functional outcomes.

**Results:**

Of 350 infants treated for any ROP, 21 (6%) were diagnosed with unilateral or bilateral AP-ROP [38 of 686 eyes (5.5%)], predominantly in zone I (77%). Infants with AP-ROP were born at a significantly younger gestational age (24.3 vs. 25.4 weeks; *p* = 0.0001) and with a lower birth weight (595 vs. 697 g; *p* = 0.0262). Treatment occurred at a significantly lower postmenstrual age (PMA) and postnatal age (PNA). Treatment consisted mainly of intravitreal anti-VEGF therapy (79% of eyes) [compared to laser photocoagulation for controls (60%)]. Eyes with AP-ROP required more retreatments (39% vs. 15%; *p* = 0.0002). At follow-up of approximately 12 months PNA, 12/15 eyes with AP-ROP had central fixation and 6/15 eyes exhibited strabismus. Median spherical equivalent of eyes with AP-ROP (*N* = 16) was 0.0 dpt (interquartile range: −2.6 to 1.0 dpt). One child developed complete retinal detachment in 1 eye and 1 child partial retinal detachment in 1 eye.

**Conclusion:**

Our study confirmed known risk factors for the development of AP-ROP such as low GA and low birth weight, whereas other previously reported risk factors such as male sex and certain comorbidities were not observed in our cohort. Our data provide additional insight into AP-ROP treatment patterns including earlier time of treatment, higher risk for retreatment, and therapy preference for intravitreal anti-VEGF therapy.

## Introduction

According to the ICROP-3 classification, retinopathy of prematurity (ROP) is classified into zones to describe the location and stages to describe the activity of ROP (1). Additionally, the so-called plus disease is characterized by dilation and tortuosity of the vessels at the posterior pole of the eye. A subtype of ROP is the aggressive retinopathy of prematurity (A-ROP), which used to be called aggressive-posterior ROP (AP-ROP) before ICROP-3 was published in 2021 (1, 2). The ICROP-3 classification now acknowledges the fact that A-ROP can also occur in bigger children and in more anterior parts of the retina and the term was therefore changed to the more inclusive term A-ROP. Since the cohort in this analysis was collected and graded before publication of ICROP-3, the term AP-ROP is used in this manuscript. AP-ROP is characterized by flat extraretinal proliferations, a circular vessel arcade at the vascularization front, as well as pronounced plus disease (2). Another very important clinical characteristic of AP-ROP is its rapid progression to tractional retinal detachment, without going through the typical stages of ROP, if treatment is not applied in a timely manner.

To date, little is known about risk factors for AP-ROP. This may be due to the fact that a relatively small number of patients develop this aggressive type of ROP, especially in developed countries (3). A multicenter analysis, like the German Retina.net ROP registry, can therefore be a valuable resource for analyzing a substantial number of AP-ROP cases. In this study, we report the results from our sub-analysis of the German Retina.net ROP registry comparing all infants treated for AP-ROP between 2011 and 2020. Our aim is to identify parameters that are associated with AP-ROP and may point to causative factors that can potentially be addressed in the prevention or treatment of aggressive ROP and report functional outcomes for patients treated for AP-ROP.

## Materials and methods

### Ethical statement

The German Retina.net ROP registry (German clinical trials register: DRKS00004522) was established in 2011. Until 2020, it was available to all ophthalmologists treating infants with ROP in Germany. In 2021, the German Retina.net ROP registry was opened for centers outside Germany and continued as the EU-ROP registry (clinicaltrials.gov: NCT04939571)^1^ (4). Between 2011 and 2020, 19 German ophthalmological centers contributed data to the German Retina.net ROP registry. The German Retina.net ROP registry is strictly observational, collecting data from routine ROP screening, ROP treatment and follow-up examinations without predefined assessments, interventions or additional examinations. Ethics approval was obtained from the leading ethics committee in Greifswald (BB 165/19) and all local ethics committees before data collection started. The study adheres to the Declaration of Helsinki. Written informed consent was obtained from the parents of all included ROP patients. The current cohort covers approximately 15% of all infants treated for ROP in Germany during the observation period (2011–2020) (5, 6). Although the number of participating centers increased steadily over time, the numbers of patients included into the registry fluctuated with no clear trend towards higher numbers [minimum 22 patients in 2011, maximum 43 patients in 2018 (7)].

### Patients

The subgroup analyzed in this study comprises all infants treated for unilateral or bilateral aggressive posterior ROP (AP-ROP) born between 2011 and 2020. This AP-ROP group was compared to infants with all other stages of treatment-warranting ROP from the same time period for: gestational age (GA), birth weight (BW), sex, initial neonatal care, postmenstrual age (PMA) and postnatal age (PNA) at ROP treatment, treatment type, need for retreatment, treatment complications, as well as neonatal parameters such as postnatal oxygen demand, and comorbidities [e.g., bronchopulmonary dysplasia (BPD), sepsis, cerebral hemorrhage, necrotizing enterocolitis (NEC) or patent ductus arteriosus (PDA)]. Infants/eyes lacking information on ROP severity at the time of treatment were excluded (*N* = 3 infants, 6 eyes).

### Statistical methods

Statistical analysis was performed using SPSS V.28 (IBM Corp., Armonk, NY, USA). Categorical variables are presented as percentages of children, continuous variables as mean and standard deviation or as median with interquartile range depending on the type of data. Due to the fact that the data analyzed in this study result from an observational registry, not all information are available for every child, and children were not excluded due to missing data. Therefore, *N* indicates the number of children or eyes for which a respective information is available. Differences between infants with AP-ROP and infants with other treatment-warranting ROP stages were calculated using unpaired Welch’s tests for continuous variables and using Fisher’s exact test for categorical variables, with a *p*-value of ≤0.05 considered statistically significant. Linear regression was used to assess changes in the prevalence of AP-ROP over time. The infants analyzed in this study were part of previously published larger analyses from the Retina.net ROP registry and studies at local centres (3, 7–12), but were not analyzed or published as a subgroup with the in-depth analysis on AP-ROP as published in this paper.

## Results

Of all infants treated for ROP in the Retina.net ROP registry between 2011 and 2020, 6% (21/350) were diagnosed with unilateral or bilateral AP-ROP, representing 5.5% (38/686) of all treated eyes. In 26 of the treated eyes with AP-ROP (68%, 26/38), information on the localization of the vascular front (zone of ROP) was available. In 77% (20/26) of eyes, the disease was located in zone I, in 23% (6/26) in zone II. The proportion of eyes treated for AP-ROP in relation to the total number of all treated eyes fluctuated from year to year between 0% (2013, 0/84 eyes) and 10% (2017, 6/60 eyes). We did not observe a trend for a general increase or decrease in the proportion of AP-ROP over the observed decade (slope −0.1170; CI: −1.053 to 0.8186; *p* = 0.7804) (Supplementary Figure 1).

**Figure 1:**
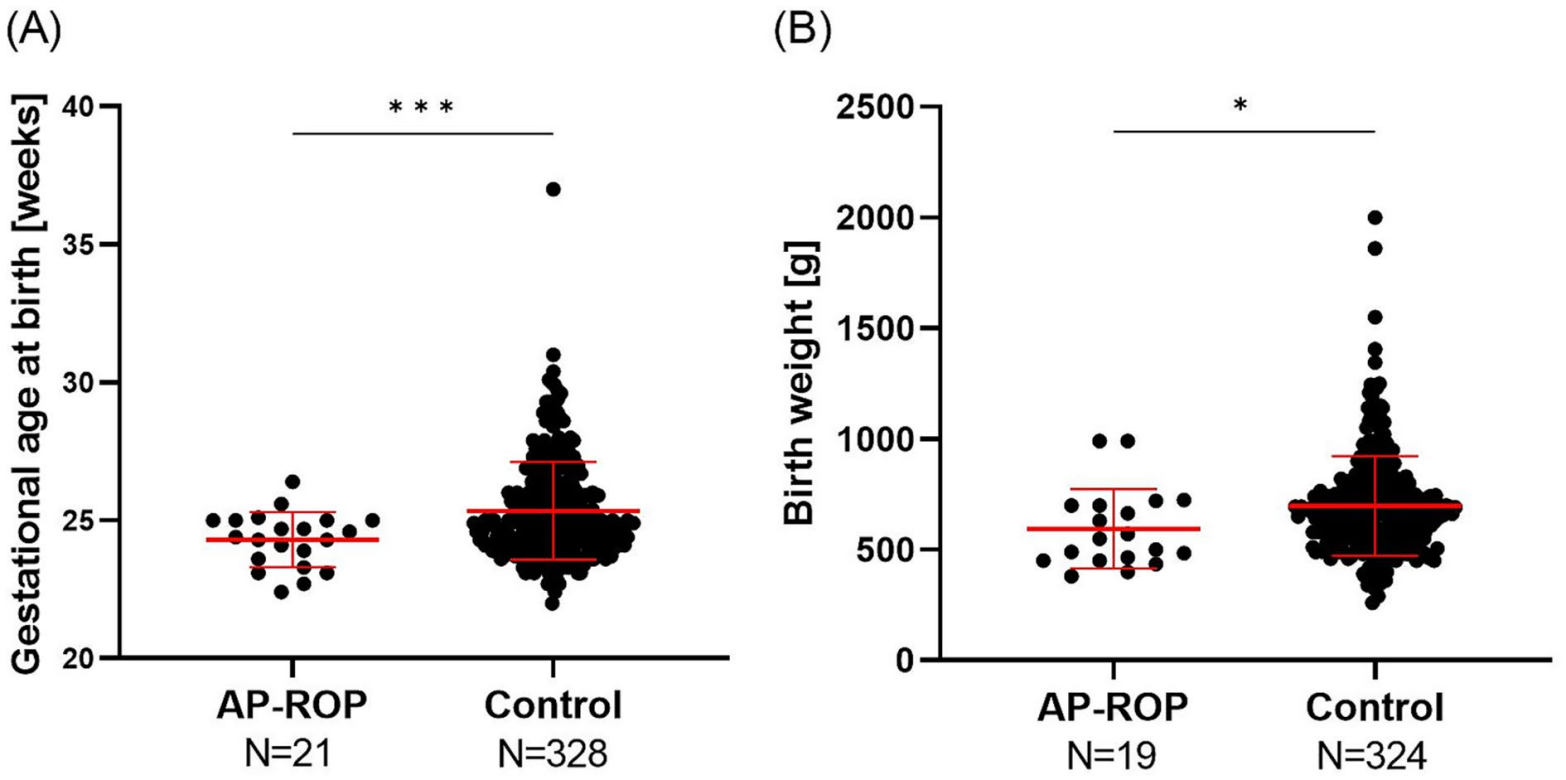
Distribution of gestational age at birth and birth weight in the AP-ROP (aggressive-posterior retinopathy of prematurity) and the control group. N gives number of patients the respective parameter is available for.

Preterm infants diagnosed with AP-ROP in at least one eye were significantly (*p* = 0.0001) younger at birth [mean GA 24.3 weeks (±1.0)] than the control group [GA 25.4 weeks (±1.8)], and had lower BW [mean 595 g (±179) vs. 697 g (±226), *p* = 0.0262; Figure 1]. The following parameters were similar for the two groups: (a) proportion of infants small for GA (BW below the 10th percentile, AP-ROP: 4/19 = 21%, control group: 48/324 = 15%, *p* = 0.5065, Fisher’s exact test); (b) male sex (AP-ROP: 15/21 = 71%, control group: 178/327 = 54%, *p* = 0.1741, Fisher’s exact test); transferal to the participating center for treatment (with initial neonatal care and ROP screening performed elsewhere; AP-ROP: 8/20 with external neonatal care = 40%, control group: 148/325 with external neonatal care = 45.5%, *p* = 0.6529, Fisher’s exact test) (Table 1).

**Table 1:** Demographic parameters of presented cohort.

Demographic parameters	Infants with AP-ROP (*N* = 21)	Infants with other ROP stages (*N* = 329)	*p*-value
Gestational age, mean [weeks] (SD) (*N* = 349)	24.3 (1.0)	25.4 (1.8)	**0.0001**
Birth weight, mean [g] (SD) (*N* = 343)	595 (179)	697 (226)	**0.0262**
Small for gestational age [*N*] (%) (*N* = 343)	4 (21)	48 (15)	0.5065
Sex [male (*N*) (%)] (*N* = 348)	15 (71)	178 (54)	0.1741
Initial neonatal care [external [*N*]) (%)] (*N*=345)	8 (40.0)	148 (45.5)	0.6529
PMA at treatment, mean [weeks] (SD) (*N* = 349)	35.2 (1.9)	37.9 (3.2)	**<0.0001**
PNA at treatment, mean [weeks] (SD) (*N* = 350)	10.9 (1.8)	12.5 (3.1)	**0.0009**
Weight at treatment, mean [g] (SD) (*N* = 157)	1,862 (724)	2,347 (701)	0.08
Weight gain from birth to treatment, mean [g] (SD) (*N* = 156)	18 (6.5)	20 (5.3)	0.38

*P*-values that are significant (≤ 0.05) are marked in bold.

**Figure 2:**
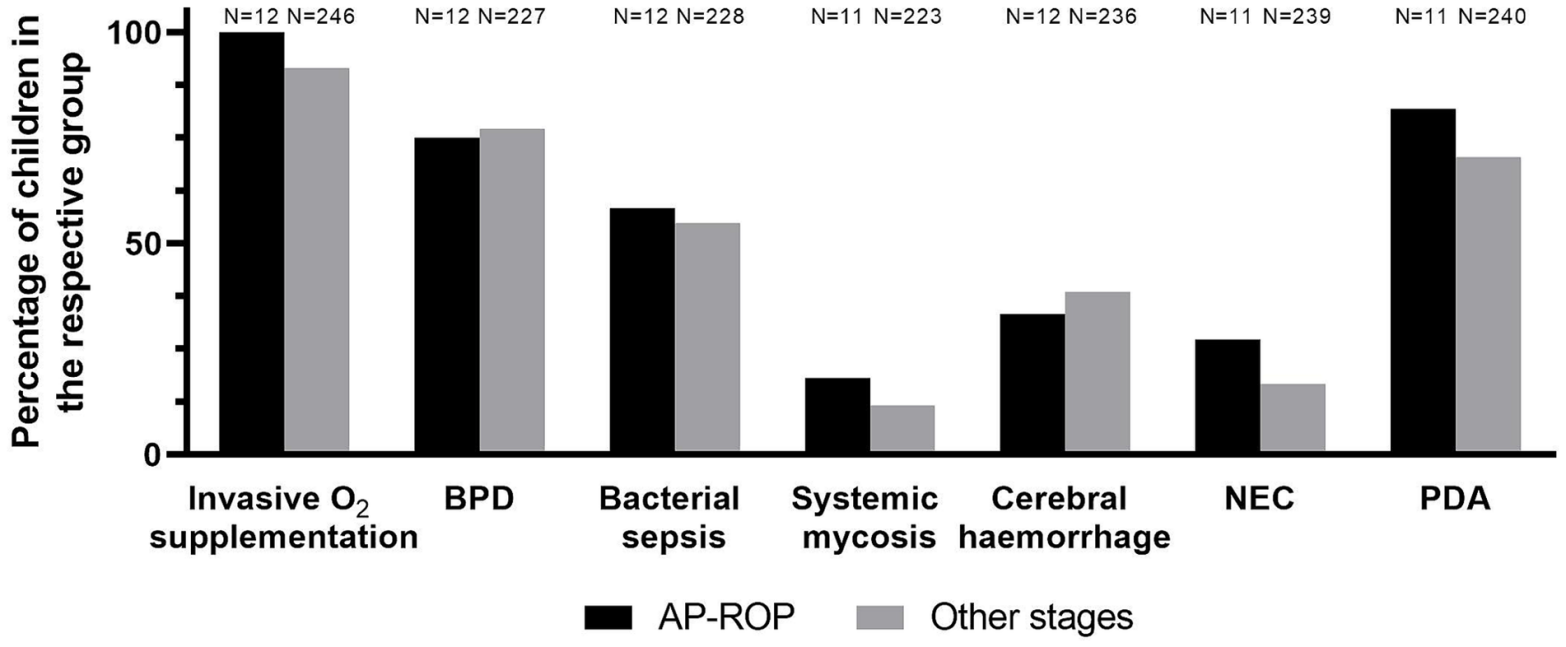
Percentage of children in AP-ROP and control group with concomitant neonatal diseases (BPD, sepsis, cerebral hemorrhage, NEC, PDA) and invasive oxygen supplementation.

Children with AP-ROP were treated at a significantly younger postmenstrual age (PMA) [35.2 (±1.9) (*N* = 21) vs. 37.9 (±3.2) (*N* = 328) weeks, *p* < 0.0001] and postnatal age (PNA) [10.9 (±1.8) (*N* = 21) vs. 12.5 (±3.1) (*N* = 329), *p* = 0.0009] compared to the control group. Weight at treatment [1,862 g (±724) (*N* = 9) vs. 2,347 g (±701) (*N* = 148), *p* = 0.08] and mean daily weight gain from birth till treatment decision [18 g (±6.5) (*N* = 9) vs. 20 g (5.3) (*N* = 147), *p* = 0.38] did not differ significantly between the two groups (Table 1). Concomitant neonatal diseases (BPD, sepsis, cerebral hemorrhage, NEC, PDA) and invasive oxygen supplementation were not different between the two groups (Figure 2).

**Figure 3:**
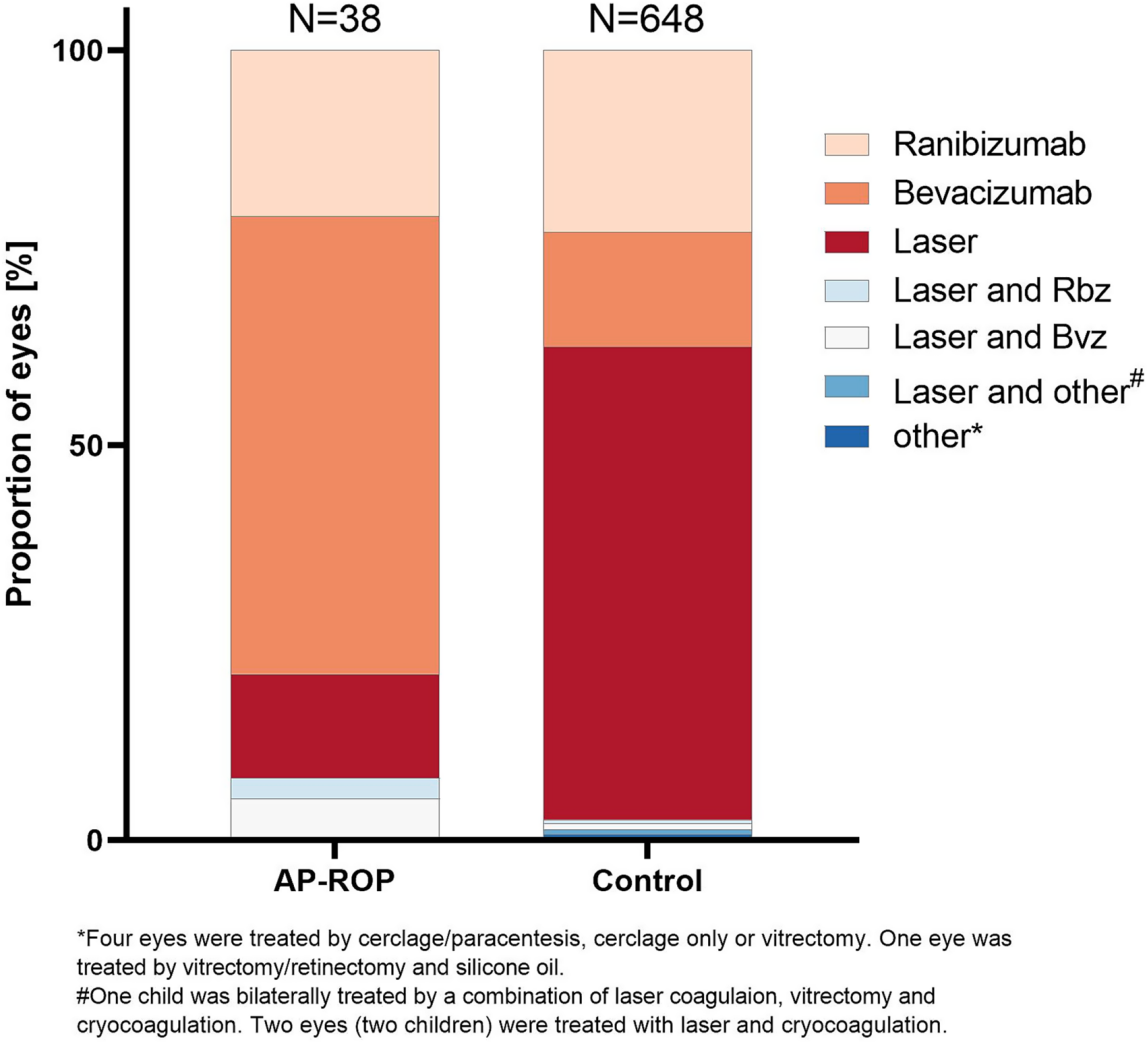
Relative distribution of treatment methods in the two groups. Almost 80% of eyes with AP-ROP were treated with anti-VEGF drugs [bevacizumab (58%), ranibizumab (21%)], while laser photocoagulation (laser) was performed in only 13% of these eyes. In the control group, bevacizumab was used in 15%, ranibizumab in 23%, and laser in 60%. A combined treatment of laser and anti-VEGF was also more common in the AP-ROP compared to the control group (8% vs. 1%).

The treatment type administered for initial treatment differed considerably between the two groups: Almost 80% of eyes with AP-ROP were treated with anti-VEGF drugs [bevacizumab (58%), ranibizumab (21%)], while laser photocoagulation (laser) was performed in only 13% of these eyes. In contrast, in the control group, bevacizumab was used in 15%, ranibizumab in 23% and laser in 60%. A combined treatment of laser and anti-VEGF was also more common in the AP-ROP compared to the control group (8% vs. 1%) (Figure 3). Among the anti-VEGF medications used for AP-ROP, a change in preference from bevacizumab to ranibizumab occurred after approval of ranibizumab for ROP in 2019 (Supplementary Figure 2).

**Figure 4:**
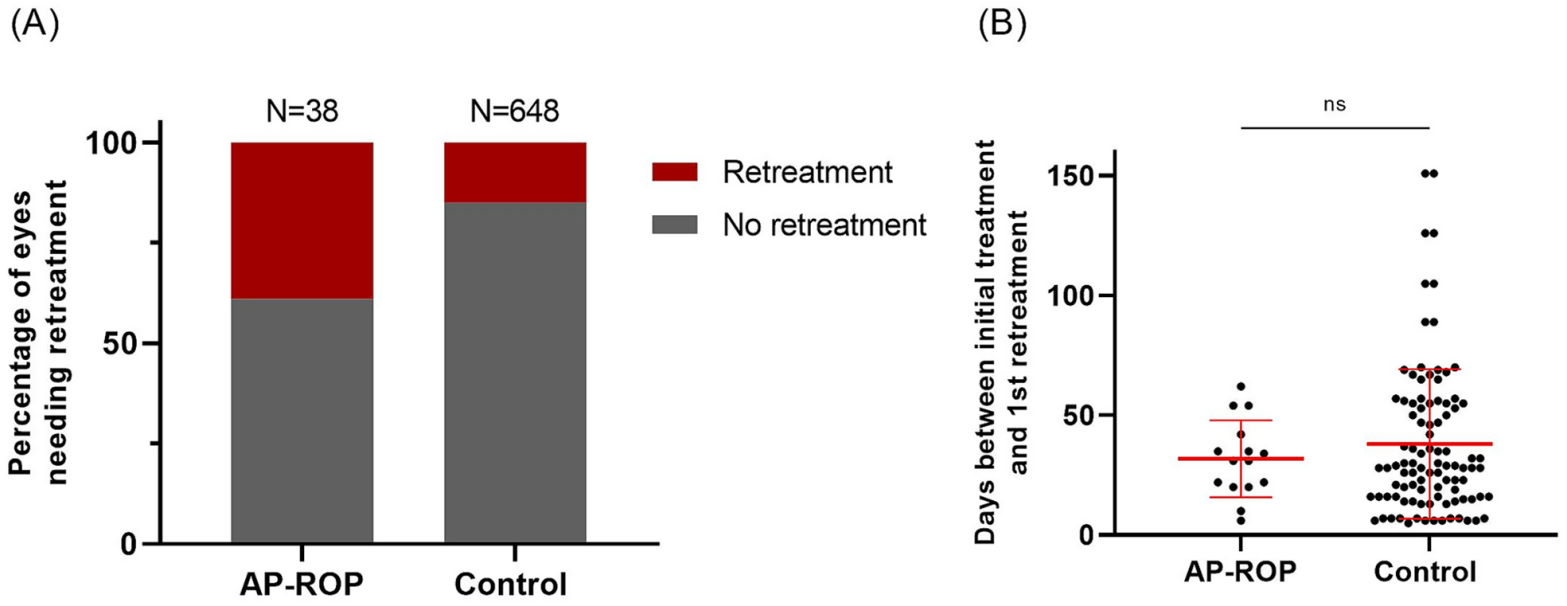
Retreatment rates in the AP-ROP and control group **(A)** and time between initial treatment and first retreatment in the two groups **(B)**. Retreatment rate was significantly higher in the AP-ROP than in the control group (*p* = 0.0002) **(A)** and tended to occur slightly earlier in eyes with AP-ROP than in the control group (*p* = 0.2434) **(B)**.

Eyes with AP-ROP required retreatment more frequently than controls (15 of 38 eyes, 39% for AP-ROP vs. 97 of 648 eyes, 15% for the control group, *p* = 0.0002; Figure 4A). The odds for the need of retreatment in eyes with AP-ROP were 3.71 times the odds in control eyes (95% CI: 1.897–7.098, *p* = 0.0004, Fisher’s exact test). Retreatment tended to occur slightly earlier in eyes with AP-ROP than in the control group, but the difference was not significant (mean: 32 days vs. 38 days; *p* = 0.2434; Figure 4B). In addition, 10/38 eyes with AP-ROP needed a second and 2/38 eyes a third retreatment, in the control group 20/648 eyes needed a second and 1/648 eyes a third retreatment. Details of the (re)treatment patterns for eyes with AP-ROP are shown in Supplementary Figure 3.

Among all infants with AP-ROP documented in our cohort, no systemic treatment complication was documented within 24 h after treatment and only in one child (left eye) an ophthalmological treatment complication within 24 h after treatment was documented. This complication was a corneal erosion treated effectively with topical antibiotics for 10 days.

Functional outcome data at approximately 1 year PN (mean 10.3 months) was available for 11 of the 21 infants with AP-ROP (Supplementary Table 2): Most eyes treated for AP-ROP had central fixation (12/15), three eyes demonstrated eccentric fixation on examination. Information as to the presence of strabismus was documented for 15 eyes (9 infants). 8 of 15 eyes had no strabismus. Two eyes/2 infants displayed intermittent exotropia, 3 eyes/2 infants had intermittent esotropia, 1 eye/1 infant a continuous exotropia and 1 eye/1 infant esotropia without documentation of intermittent or continuous. Median spherical equivalent of all AP-ROP eyes (*N* = 16) was 0.0 (IQR: −2.6 to 1.0) diopters. One child (#1) had developed complete retinal detachment in one eye together with severe macular traction, and another child (#2) mild partial retinal detachment in one eye not involving the macula. One child (#6) had a moderate macular traction and moderate peripheral pigment endothelial alterations resulting from laser scars, and one child (#7) had in both eyes a mild optic nerve atrophy, in the right eye mild macular tractions and moderate central pigment epithelial alterations and in the left eye moderate peripheral pigment epithelial alterations. No reliable visual acuity data were available at this young age. For more information of functional results, all data documented at a follow-up visit of infants with AP-ROP up to a maximum age of 6 years and 3.5 months are displayed in Supplementary Table 2. One child (#1), who had severe macular traction and complete retinal detachment in the right eye at about 1 year of age, developed moderate glaucoma by the age of 31 months. Child #7 was diagnosed with severe visual impairment at the age of 40 months. In general, we observed more ocular findings documented at any of the post-operative follow-up assessments in the AP-ROP group than in the control group (*N* = 38 post-operative follow-up assessments documented in the 21 infants with AP-ROP and *N* = 648 in 329 infants in the control group) (e.g., optic nerve atrophy 15.8% vs. 3.7%, retinal detachment 7.9% vs. 1.7%, macular traction 7.9% vs. 0.8%; see Supplementary Figure 4).

## Discussion

The most aggressive form of ROP, formerly known as AP-ROP, now referred to as A-ROP, is dreaded by all ophthalmologists involved in the management of ROP because of its risk of rapid progression to tractional retinal detachment and severe visual impairment. Its fast progression increases the risk of being missed and is a key feature of this most aggressive form of ROP (1). Little is known about risk factors for the development of this aggressive subtype of ROP or its accelerated progression. In this study, we analyzed all infants with treatment-warranted ROP from the German Retina.net ROP registry (2011–2020) to elucidate the characteristics of AP-ROP.

Key findings were: (1) extremely preterm infants with the lowest GA and BW had the highest risk for AP-ROP; (2) AP-ROP incidence remained similar from 2011 to 2020; (3) AP-ROP eyes were treated at an earlier PMA and PNA compared to eyes with other stages of ROP; (4) most AP-ROP eyes received anti-VEGF alone or in combination with laser as initial treatment; (5) AP-ROP eyes had 3.71 times higher retreatment odds.

AP-ROP affects only a minority of preterm infants. In our cohort, only 6% of infants with treatment-warranted ROP were diagnosed with AP-ROP. While this proportion is similar to data from the Swedish SWEDROP registry (8.3%) (13), an Australian dataset (12.1%) (14) and a Polish cohort (10.4%) (15), AP-ROP rates as high as 24.2% have been reported from a Korean cohort (16). These differences in AP-ROP rates may reflect differences in population and neonatal care (17), but could also be a statistical peculiarity based on the overall low numbers of this subtype of ROP (see Supplementary Figure 1). Another reason for the differences may be the fact that AP-ROP can be difficult to diagnose especially when treating early and when fluorescence angiography is not available (11). It has already been described from other countries, that AP-ROP typically affects preterm infants with a very low GA and/or very low BW (14, 16, 18). In Germany, due to highly developed neonatal care, almost all infants who develop any stage of treatment-warranted ROP are extremely preterm and have a very low birth weight (7). One question in this study was therefore whether, even in this cohort of overall extremely preterm infants, differences in GA and/or BW were still evident between infants with AP-ROP and other types of treatment-warranted ROP. Our data demonstrates that even in such a cohort of overall extremely preterm infants, those with the lowest GA and BW were those with the highest risk of AP-ROP. The mean GA of 24.3 weeks and the mean BW of 594 g observed in our AP-ROP cohort are in good agreement with those reported in Australian (14) and US cohorts (18).

In contrast to other studies (16, 19), we did not find intrauterine growth restriction resulting in small for gestational age (SGA) infants or postnatal daily weight gain as a surrogate parameter for postnatal development to be associated with AP-ROP. However, we did observe that infants with AP-ROP underwent treatment earlier, both in terms of PMA as well as PNA. This is consistent with results from Ahn et al. (16) and Bellsmith et al. (18) who reported an about 2 weeks younger PMA at treatment in children with AP-ROP compared to children with other forms of treatment-warranted ROP.

Neonatal comorbidities such as chorioamnionitis, multiple infections and sepsis have been described as risk factors for the development of AP-ROP (16, 19, 20). Other groups, however, have not confirmed associations of AP-ROP with NEC or sepsis (18). The influence of lung diseases such as respiratory distress syndrome or bronchopulmonary dysplasia is also controversial (16, 18, 19, 21). In our cohort, comorbidities were not statistically different between children with AP-ROP and the control group (see Figure 2). However, the study is underpowered to rule out modest associations with sepsis, NEC, etc.

We found the biggest difference between our cohort of AP-ROP and other forms of treatment-warranted ROP regarding the type of treatment. Anti-VEGF therapy predominated over laser in AP-ROP eyes (anti-VEGF 79% vs. laser 13%), whereas laser was more frequent in eyes with other types of ROP (38% anti-VEGF vs. laser 60%). This is probably due to the fact that anti-VEGF is often chosen for the most central and rapidly progressing disease stages, as seen in other analyses from the Retina.net ROP Registry (3, 7). In a meta-analysis, Wang et al. (22) concluded that anti-VEGF therapy is as effective regarding the prevalence of recurrences and the need for retreatments, and is more beneficial than laser therapy for the treatment of type 1 ROP and AP-ROP regarding high myopia and the rate of complications.

The significantly higher retreatment rate for AP-ROP compared to controls in our cohort (39% vs. 15%) is consistent with other studies (19, 23–26). Retreatment rates after anti-VEGF exceed those after laser (25, 26), possibly contributing is the fact that most AP-ROP eyes received anti-VEGF and other ROP eyes predominantly laser therapy as initial treatment. Interestingly, the risk for retreatment was significantly higher in eyes with AP-ROP who had received laser therapy as initial treatment (80%, 4/5 eyes) compared to eyes with AP-ROP who had received anti-VEGF therapy as initial treatment (30%, 9/30 eyes) (see Supplementary Figure 3). Due to the small number of children treated with laser alone and the nature of real-world registry data, potential confounders cannot be excluded and further studies with larger numbers are needed. A study of Narnaware et al. (27) on eyes with AP-ROP treated with laser reported a retreatment rate (additional laser applied within one to 2 weeks after initial laser treatment) similar to the one we report for anti-VEGF treatment (35.7%; 20 of 56 eyes).

Regarding long-term outcomes, unfavorable structural outcomes were more frequent in children with AP-ROP compared to the control group. It is well described that unfavorable structural outcomes after treatment-warranted ROP are common (1). But due to differences in inclusion criteria and reported ages as well as definitions of unfavorable outcomes it is difficult to compare rates among studies. In addition, our study may have some degree of observer bias as patients with AP-ROP are more often intensively followed-up than patients with other stages of ROP, which might result in a higher rate of ocular findings in the group with more frequent follow-ups. Therefore, more detailed, large-scale analyses of long-term data are needed. One of the strengths of our study is that it is a real-world registry study with data from 19 centers now transitioned to the European EU-ROP registry (see text footnote 1), which will provide larger data sets for analyses in the near future.

One of the limitations of this study is that the current cohort represents only about 15% of all infants treated for ROP in Germany during the observation period. Therefore, the results may be influenced by selection bias and may not fully capture regional or center-specific treatment variations, thereby potentially limiting the generalizability of the findings. Another limitation is that details of oxygen supplementation were documented very sparsely in the Retina.net ROP registry despite the fact that oxygen therapy is known to play a major role in the development of A-ROP (28). The fact that certain parameters are not documented for every child (e.g., weight at treatment) and the different group sizes are also limitations.

## Conclusion

In summary, this real-world registry analysis confirms known risk factors for AP-ROP including low GA and BW. In addition, it provides novel insights into treatment patterns of these infants, i.e., earlier time of treatment, higher risk for retreatment and therapy mainly with intravitreal anti-VEGF. Other proposed risk factors like male sex or association with specific comorbidities were not confirmed in this cohort. Given the overall rarity of AP-ROP, a multicenter approach like the one presented here is pivotal. However, our sample size is still limited. Expanding collaboration through registries like the now established EU-ROP registry will enable more robust research in the field of this rare subtype of ROP. Therefore, we encourage all centers that treat ROP across geographical Europe to contribute to the registry in order to support the generation of high-quality datasets for future analyses (see text footnote 1).

## Data Availability

The datasets presented in this article are not readily available because of data protection reasons. Requests to access the datasets should be directed to johanna.pfeil@med.uni-greifswald.de.

## Footnotes

1
www.eu-rop.org

## Group member of German Retina.net ROP Registry Study Group

Retina.net ROP registry study group members are listed in Appendix 1.
